# Radiation-free assessment of the 3D morphology of the adolescent scoliotic spine: a feasibility study using MRI derived quantitative synthetic CT

**DOI:** 10.1007/s43390-026-01399-7

**Published:** 2026-05-13

**Authors:** Lorenzo Costa, Peter Lafranca, Tijl A. van der Velden, Noah Allaerts, Rutger A. J. Nievelstein, René M. Castelein, Tom P. C. Schlosser, Peter R. Seevinck

**Affiliations:** 1 Department of Radiology & Nuclear Medicine, Division Imaging & Oncology, University Medical Centre Utrecht, Utrecht, the Netherlands; 2 Department of Orthopaedic Surgery, University Medical Centre Utrecht, P.O. Box 85500, Utrecht, 3508 GA the Netherlands; 3 MRIguidance BV, Utrecht, The Netherlands

**Keywords:** Synthetic CT, SCT, Adolescent idiopathic scoliosis, BoneMRI, Cobb angle

## Abstract

**Introduction:**

Treatment management of pediatric spinal deformity patients heavily relies on radiological imaging; always with X-rays and sometimes CT scans to better delineate 3D morphology, and increasingly for navigation purposes. It is important to minimize radiation exposure in this vulnerable pediatric population, since it increases the risk for cancer development. This pilot study investigates the feasibility and reliability of 3D-spinal parameters identified on MRI-based quantitative synthetic CTs (sCTs) to assess scoliosis in adolescents.

**Methods:**

Ten pre-operative MRI’s from adolescent idiopathic scoliosis patients (mean age: 13.5 years) with curves on standing x-ray > 40° were included. A 4-min 3D spoiled gradient echo MRI sequence capturing the main thoracic curve was added to the clinical sequences and quantitative sCTs were generated. Three independent observers measured Cobb angle, T4-T12 thoracic kyphosis (TK) and apical vertebral rotation (AVR). Interrater reliability and 95% confidence intervals (CI) were calculated.

**Results:**

The sCTs clearly visualized the vertebral bodies and posterior elements, allowing for easy landmark identification to assess spinal morphology parameters of individual discs and vertebral bodies. Minor artifacts were observed in intervertebral discs and near the respiratory tract, not affecting the landmark positioning. The interrater reliability ICCs were Cobb angle: 0.99 (95% CI 0.98–1.00), TK: 0.98 (0.95–1.00) and AVR: 0.95 (0.86–0.99).

**Conclusion:**

This pilot study confirms that sCT derived from MRI allows for radiation-free and reliable measurement of 3D deformity parameters in AIS patients. While further research is needed, these findings demonstrate the potential for high-quality MRI-based bone imaging and its possible application in AIS treatment planning.

## Introduction

Adolescent idiopathic scoliosis (AIS) is a common 3D spinal deformity affecting 2–4% of the general population [1–3]. During the adolescent growth spurt the spinal and trunk deformation can progress rapidly and this is influenced by multiple factors such as sex, skeletal maturity, curve type and curve magnitude at diagnosis [1, 2, 4]. To allow for effective management, regular follow-up visits are crucial for clinical examination and to assess the radiographic curves’ magnitude and skeletal maturity [5–7].

Diagnostic imaging plays a pivotal role in scoliosis management; mostly conventional radiography, biplanar radiography, bending and/or traction studies and full-spine MRI. Full-spine radiographs are normally acquired in the standing position to detect the coronal magnitude of the scoliotic deformity, measured via the Cobb method, as well as sagittal alignment [8]. As radiographic imaging allows fast and readily available images, it is widely used initially for screening of non-idiopathic causes and for follow-up of curve progression in scoliotic patients [9]. Nevertheless, conventional radiographs provide 2D-projections, and they do not show the characteristic complexity of the axial plane spinal deformity as well as the deformation of the non-osseous structures such as the discs. Recently, it has become more apparent that the primary deformity in AIS starts in the IVDs, as compared to the bone [10–13].

In addition to radiographs, full spine MRIs are often obtained in patients with more severe AIS for screening of neural axis abnormalities and other soft tissue pathologies preoperatively. When the curve magnitude increases despite conservative treatment, surgery is usually indicated [5, 7]. With the advancements of surgical navigation and robotic surgery, nowadays, also preoperative CTs and intra-operative 3D images are obtained regularly for preoperative planning and/or to visualize the critical landmarks for pedicle screw instrumentation. Of course, both CT as well as intra-operative 3D imaging requires ionizing radiation and should be avoided as much as possible in adolescents because of the small but non-negligible risk of ionizing radiation induced cancer later in life [14–17].

Ideally, a single image acquisition technique would allow for the visualization of the curve etiology, regional curve severity as well as the local 3D deformation of the osseous as well as non-osseous spinal structures in different areas of the curve. In the recent years, MRI-based image synthesis techniques based on the use of convolutional neural networking algorithms have been developed and validated, successfully generating synthetic CT scans (sCT) [18–20]. These sCTs have the potential to be combined with other MRI sequences allowing to visualize in one scanning session both bony structures and soft tissues involved in the scoliotic deformity, paving the way to new radiation-free approaches for scoliosis management and surgical planning. The sCT has already been validated and CE-marked/FDA approved for several anatomical areas, including pelvis, lumbar and cervical spine and in different age groups [18, 20–22]. In this study, the aim is to investigate the feasibility and reliability of 3D-spinal parameters identified on sCT derived from MRI scans to assess spinal deformation in AIS patients, highlighting a new way for accurately capturing the complex 3D deformity and possibly supporting management of AIS patients that require brace or surgical treatment.

## Methods

The Standard for Reporting of Diagnostic Accuracy (STARD) checklist was followed in this study [23]. This retrospective observational study qualified for an exemption from review from the Institutional Review Board because of minimal risk and burden for the patients. Patients and parents provided written informed consent before the MRI scans were obtained. Ten girls (between 12 and 16 years of age), scheduled to undergo a preoperative MRI scan for idiopathic scoliosis between November 2021 and December 2022, underwent an additional MRI sequence. Demographics were collected and coronal Cobb angle and thoracic kyphosis were measured on the most recent standing full-spine radiographs, obtained within 6 months before the MRI examination.

### Image acquisition

The examination was performed on a 1.5 T MRI (Philips Healthcare, Best, NL) and included a 3D dual echo Spoiled Gradient Echo sequence with TE1/TE2/TR = 2.1/4.2/7.0 ms, FOV 240mmx240mmx100mm, reconstructed voxel size 0.7mmx0.7mmx0.9 mm and a total acquisition time of 4m16s. The FOV was centered on the primary, main thoracic curves and included the anterior and posterior elements of the apical as well as the neutral zones. These images were processed into sCTs using BoneMRI version 1.7 (MRIguidance BV, Utrecht, the Netherlands).

### Image analysis

RadiAnt DICOM viewer 2022.1.1 (Medixant, Poznan, Poland) was utilized to reconstruct the coronal, sagittal and axial plane sCT images. These were assessed for measurement of the coronal Cobb angle, sagittal T4-T12 angle and axial apical rotation of the major curve by three trained observers, executing the analyses independently. Cobb angles were measured using the standard Cobb method on the coronal plane image where the spine was best visible, and kyphosis angle was measured using the upper endplate of T4 and the lower endplate of T12 on the sagittal plane images. In-house developed software (ScoliosisAnalysis 4.1) was used for analysis of the apical vertebral rotation. The 3D orientation of superior and inferior endplates of the apical vertebra and the first neutrally rotated vertebra were defined on multiplanar reconstructions for analysis of the Apical Vertebral Rotation (AVR). The true transverse section of the upper and lower endplates and spinal canals enabled to detect the true anterior–posterior (AP) axis of the apex [24]. AVR was defined as the angle between AP axis and the AP axis of the first distal neutrally rotated vertebra.

### Statistical analysis

A statistical analysis was performed using SPSS 22.0 (SPSS, Inc., Chicago, IL, United States). Descriptive baseline statistics for the demographics were calculated, providing mean and standard deviation (SD). Before testing, normality of distribution of the curve severity was verified using Q-Q plots, which showed that the sample was normally distributed. Intraclass correlation coefficients (ICC) and 95% confidence interval (95% CI) were calculated for assessment of interobserver reliability between the observers. To test the consistency with the existing literature on assessment of scoliosis severity in different body positions, the supine sCT Cobb-angle measurements were compared with a previously validated equation to estimate the supine Cobb angle from the upright Cobb (*2.9* + *0.79* × *Cobb in standing position*) using R^2^ [25]. Agreement between standing radiograph and supine MRI Cobb angles was assessed using Bland–Altman analysis (mean difference and 95% limits of agreement). Linear regression was used to model the relationship between MRI and X-ray measurements and identify bias [26].

## Results

The ten female patients had a mean age of 13.5 years (SD ± 1.4), a mean Cobb angle of 53° (SD ± 7.4) and a TK of 28° (SD ± 3.6) on standing x-ray. Five patients (50%) exhibited scoliotic curves with Lenke Type I and five (50%) with Lenke Type III. No patient encountered any adverse events during the procedure and all images were included in the dataset.

**Fig. 1:**
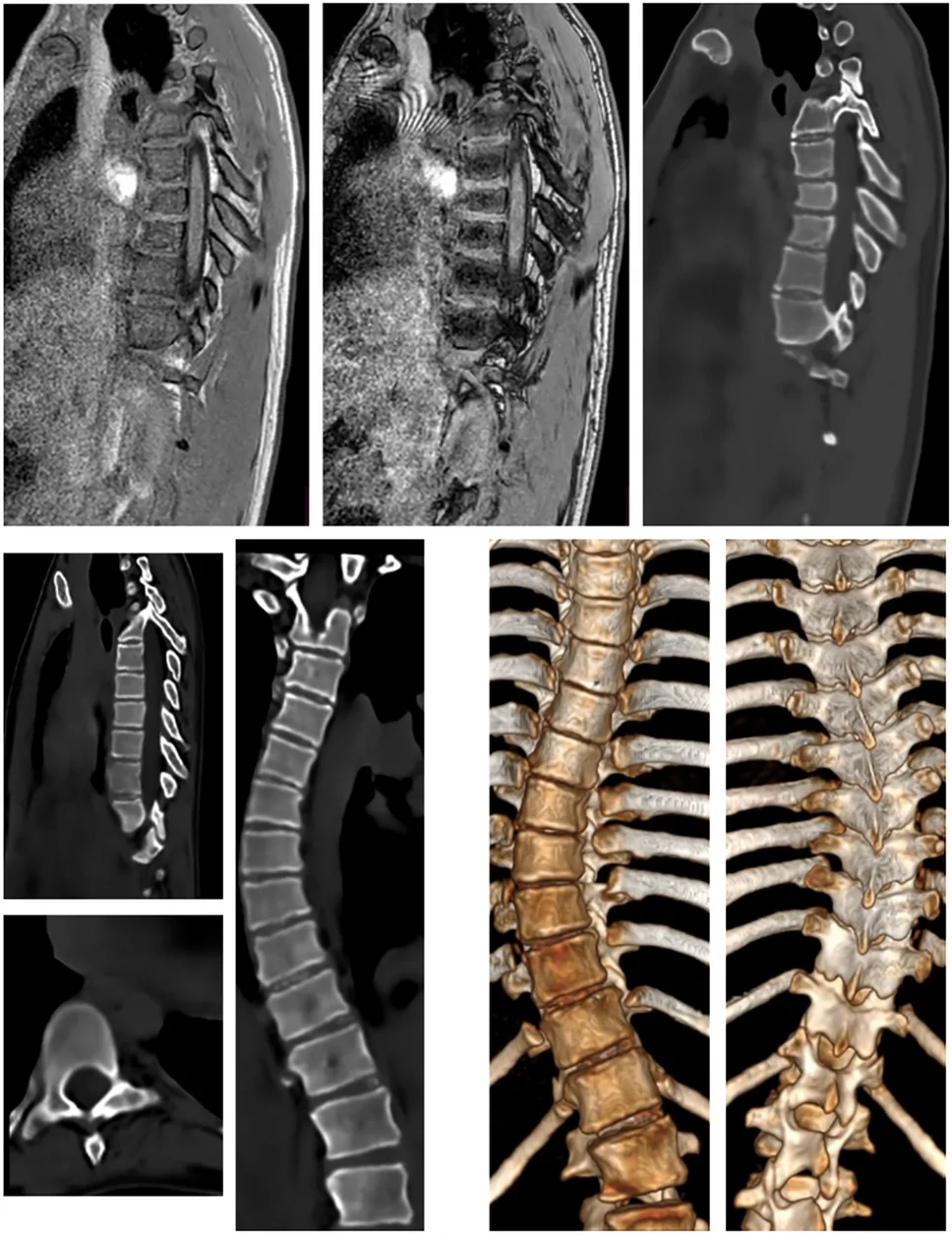
Visualization of the curved section of the spine in an MRI based sCT in a 16 year old female. In phase and out phase BoneMRI sequences (top left) from which the sCT was reconstructed (top right) can be seen on the upper part of the image. On the bottom side, multiplanar reconstruction (left side) visualizes the sagittal plane (mid left), axial plane (bottom left), and coronal plane (middle-left). The right images represent the 3D reconstruction gathered on RadiAnt (Medixant, Poznan, Poland) with a view from anterior (middle-right) and posterior (right) of the spinal deformity constructed via sCT

The sCTs provided high resolution 3D CT-like images, allowing for multiplanar and 3D reconstructions, clearly visualizing the deformed spine, effectively visualizing each vertebral body, the posterior elements as well as the costovertebral joints (Fig. 1).

**Fig. 2:**
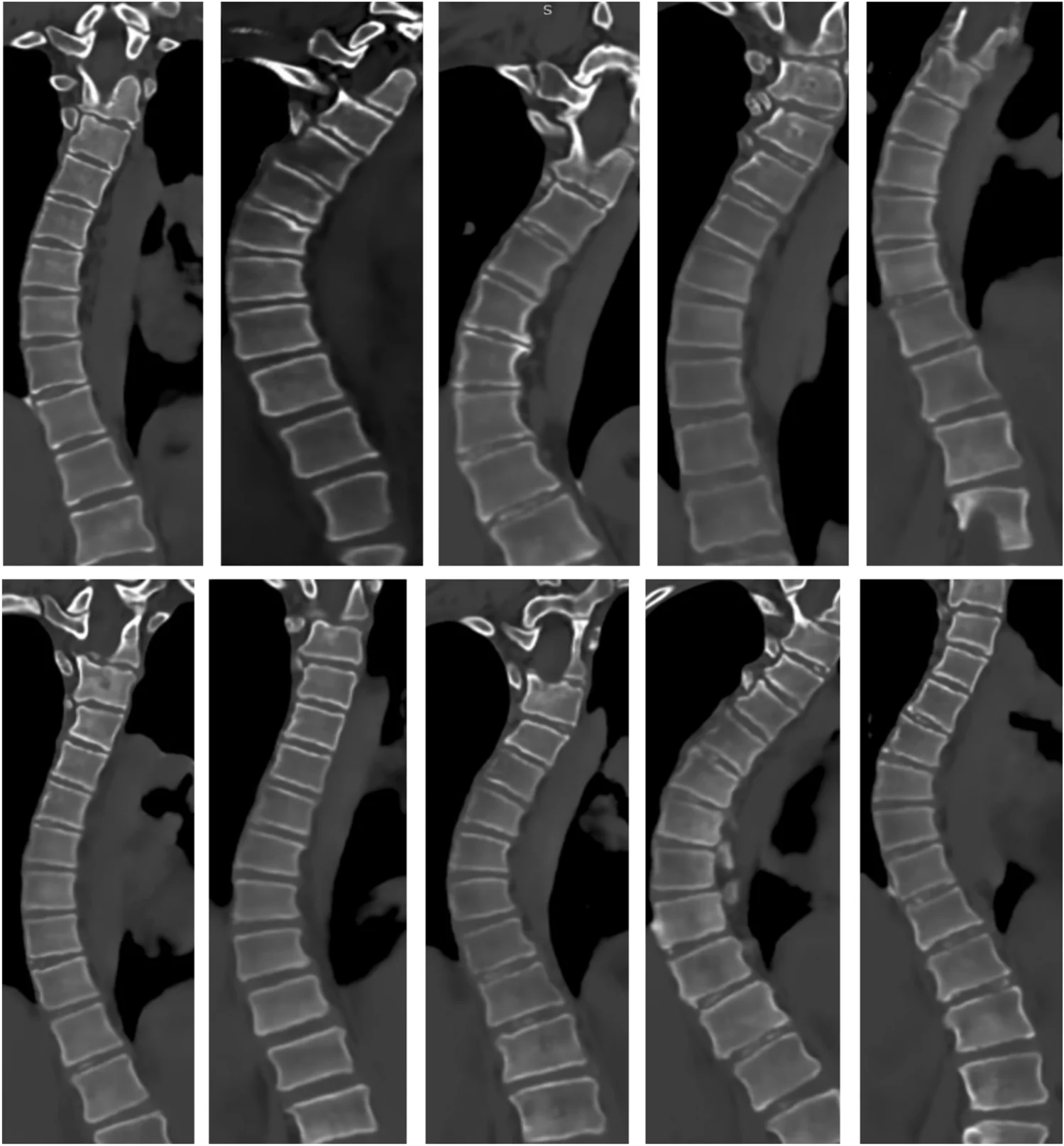
Visualization of the coronal view of the sCTs of the ten patients

Figure 2 presents coronal sections for each of the ten patients, clearly delineating the cortical bone. In some patients, hyperintensities were observed within the intervertebral discs and near the respiratory tract.

**Fig. 3:**
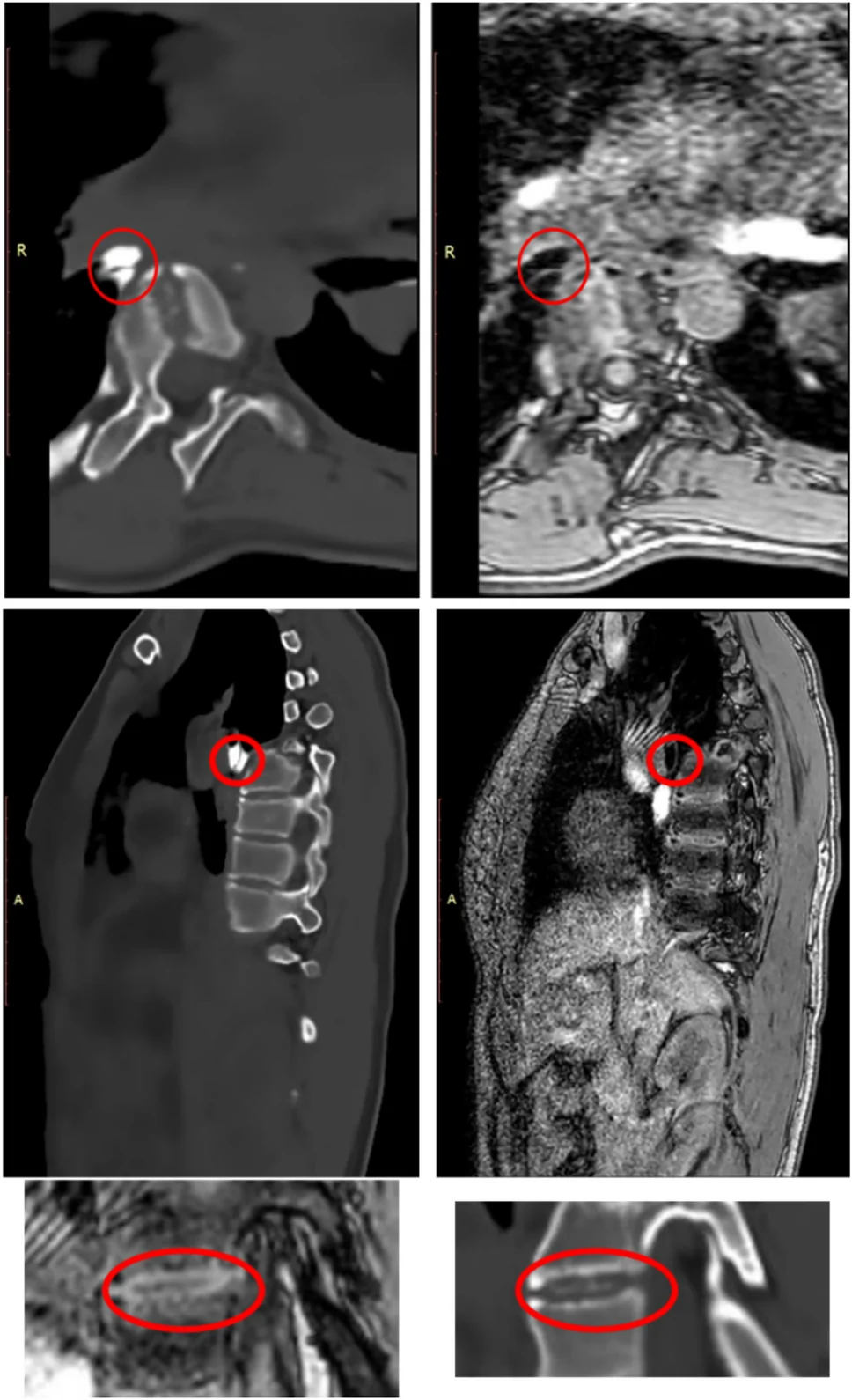
Visualization of the axial (above) and sagittal (below) views of the same patient in source data with the BoneMRI (right) and the CT-like derived by the BoneMRI sequence (left). Encircled in red can be seen that the areas of hyperintensity found on the sCT (left) correlate with areas of hypointensity in the BoneMRI sequence (right) suggesting that these areas are artifacts. Below can be seen the hypointensity areas present in the disc on BoneMRI (left) and the correspondent hyperintensity area on sCT (right)

When comparing with the MRI source data, these hyperintensities coincided with low signal areas on BoneMRI sequence, which suggests the presence of artifacts (Fig. 3).

**Fig. 4:**
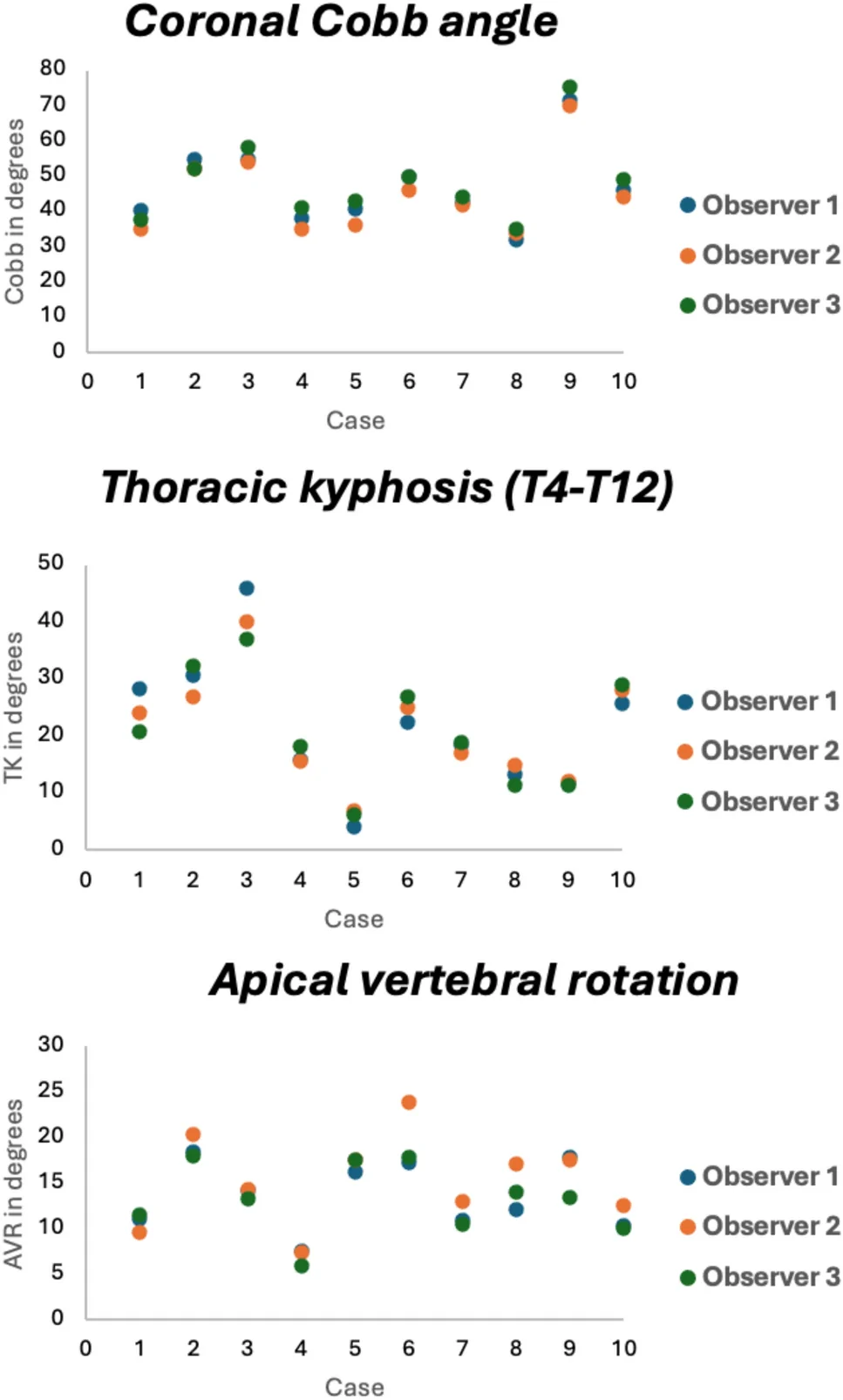
Scatter plot of the analyses performed by the three observers in terms of Cobb angle, TK, and AVR

All images were successfully analyzed by the three independent observers. A detailed comparison of the observers' assessments is presented in Fig. 4.

**Fig. 5:**
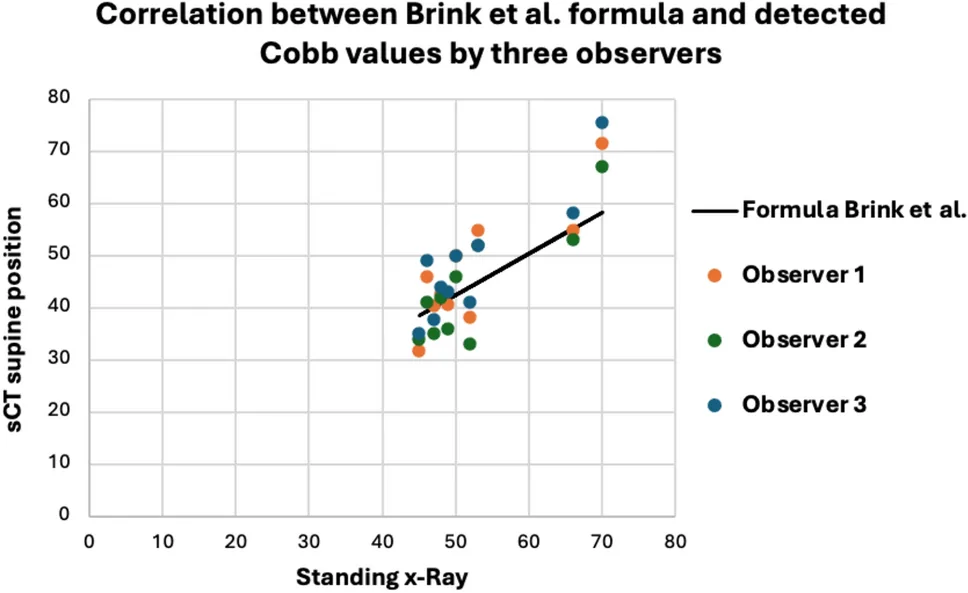
Scatter plots showing the relation between the Cobb angle in the upright and supine position measured by the three observers and the supine Cobb angle predictions calculated using the formula by Brink et al. [25]. This equation acts as a translator, using the relationship between Cobb angles measured in upright X-rays and supine MRIs. It takes the Cobb angle from a supine MRI and estimates what it might be in a standing X-ray

The ICCs for interrater reliability were 0.99 (95% CI 0.98–1.00) for Cobb angle, 0.98 (0.95–1.00) for TK and 0.95 (0.86–0.99) for AVR. Moreover, Fig. 5 demonstrates the relationship between standing and supine Cobb angle measurements, as well as the previously validated linear correlation (2017) [25]. R^2^-values for the correlation between the observers' measurements and the formula’s predictions were 0.72, 0.73, and 0.79 for observers 1, 2, and 3, respectively [25].

Bland–Altman analysis revealed a mean difference (MRI − X-ray) of − 8.2° (SD 1.8°), with 95% limits of agreement from − 11.8 to − 4.6°, indicating systematic underestimation of Cobb angle on MRI. Linear regression demonstrated a strong relationship (MRI = 2.84 + 0.79 × X-ray; R^2^ = 0.998; p < 0.001), with increasing underestimation for larger curves.

## Discussion

The current study explores the feasibility and reliability of using 3D-spinal visualizations and parameters identified on sCTs derived from MRI scans for the assessment of scoliotic deformity in AIS patients. This innovative technique offers the opportunity to assess the complex 3D spinal deformity of the osseous as well as non-osseous spinal structures in great details, without the use of ionizing radiation, in single examination.

Three observers analyzed ten sCTs from female patients undergoing pre-operative MRI scans, measuring widely used parameters such as Cobb angle, TK and AVR. Notably, inter-observer analysis demonstrated high agreement across all measurements, aligning with existing literature and supporting sCTs’ accuracy compared to standard CT scans [25]. This further highlights the potential of sCT as a reliable alternative to traditional imaging techniques, potentially reducing dependence on methods involving ionizing radiation such as standard CTs.

While some areas with hyperintensities were observed within the intervertebral discs and near the respiratory tract, the cortical bone reconstruction and overall image quality allowed for accurate measurements and these findings did not impact the image analysis, as evidenced by the high agreement between the three observers. The acquisition time of the acquired MRI, including its conversion to sCT is longer when compared to conventional CT scans, as is generally known. The sCT however, shows low noise levels and none had significant movement artifacts [20, 22]. Furthermore, this sCT technique facilitates the generation of 3D reconstructions without timely manual interaction by simply setting a threshold, as shown in Fig. 1 and Fig. 2, presenting the intricate 3D scoliotic deformity.

Currently, CTs and X-rays remain common tools for scoliosis management, but they pose a risk of DNA mutations and potentially causing various cancers due to ionizing radiation exposure [14, 27–29]. This concern is particularly relevant for adolescent females who undergoes pre-op and intra-op CTs for planning and navigation, whose developing bodies, especially the reproductive system and breast, are more vulnerable [16]. Therefore, the development and implementation of alternative radiation-free imaging techniques to visualize bony structures in scoliosis is critical to improve patient management.

Currently, other modalities, like biplanar radiography, low-dose CT, 3D spinal ultrasound and surface scanning are also being explored [29, 31, 32]. While biplanar radiography has the advantage that it is acquired in the standing position, the 3D reconstructions are virtual 3D models based on manually identified anatomical bony landmarks on the radiographs. This modality as well as low-dose CT are not radiation-free, and they provide the full 3D nature of the deformity, especially the intervertebral discs, only indirectly. Like biplanar radiography, 3D spinal ultrasound and surface scans can also be obtained in the standing position, allowing for simultaneous assessment of the coronal, sagittal and axial plane, enabling quantification of both sagittal alignment and 3D deformation [30, 31]. These radiation-free techniques, however, only visualize the posterior skin and the posterior elements, and therefore unlike sCT, also do not capture the full 3D nature of scoliosis. Furthermore, the technique presented in this study includes quantitative synthetic CTs, which in contrast to CT-like MRIs, correlates with bone density which can be of most relevance for pediatric spinal deformities in non-ambulatory patients and adult spinal deformities [33]. Table 1 shows the advantages and limitations of the imaging techniques employed.

**Table 1:** Comparision of the imaging techniques employed

Imaging modality	Position	Radiation dose	Curve magnitude assessment	Key advantages	Key limitations	Typical clinical role
Standing Conventional radiography (CR)	Standing/weight-bearing	Low	Gold standard for curve magnitude and global spinal balance	Fast, inexpensive, minimal radiation dose, universally available, 2D visualization	No 3D detail, poor visualization of vertebral anatomy and soft tissues	Routine scoliosis assessment, follow-up, and for surgical planning
Standing EOS	Standing/weight-bearing	Very low (lower than CR)	Excellent assessment of curve magnitude and global alignment	Ultra-low radiation dose, full-body alignment analysis, 3D modeling	Not widely available; specialized equipment	Longitudinal deformity monitoring and advanced alignment analysis
Supine MRI	Supine	None	Less accurate for true deformity magnitude due to supine position	No radiation, excellent visualization of discs, spinal cord, and neural elements	Supine positioning; slower and more expensive than CR and EOS	Neuraxis evaluation, disc pathology, pre-operative neurological assessment
Standing MRI	Standing/weight-bearing	None	Potentially very accurate since deformity is imaged under load	Combines weight-bearing assessment with soft-tissue visualization	Rare, expensive, long acquisition times	Emerging technology for functional spinal imaging
Supine CT	Supine	Highest radiation dose	Limited for Cobb assessment due to supine positioning	Superior 3D bone detail; ideal for pedicle screw planning and navigation	High radiation dose; less expensive than MRI; supine positioning; not suitable for routine deformity monitoring	Surgical planning, complex anatomy, navigation guidance
sCT	Supine (from MRI dataset)	None	Limited for Cobb assessment due to supine positioning	Provides bone detail with quantitative values, CT-like in Hounsfield units without radiation; simultaneous neuraxis and disc evaluation	Potential artifacts; limited availability; supine positioning;	Emerging tool for surgical planning and navigation guidance without radiation

While sCT primarily focuses on visualizing bony structures, MRI scans (the source of sCT data) can also visualize the IVD, neural elements and other soft tissues using specific sequences [34]. Research suggests that IVD plays a significant role in the biomechanical development of AIS, contributing to curve progression [10, 35, 36]. Therefore, utilizing MRIs could potentially provide a more complete picture of the 3D deformity by including both hard and soft tissue detection in one examination, limiting the burden of multiple imaging modalities to patients and need for image fusion techniques. Furthermore, in navigation surgery the asymmetric position of the spinal cord can be considered.

Standard CTs are often used in young patients with scoliosis for pre-operative planning and intra-operative navigation surgery. However, as previously discussed, this technique relies on ionizing radiation, which carries potential risks related to DNA damage [6]. sCT offers a promising alternative as it has the potential to capture complex bony structures as well as soft tissues such as intervertebral discs and, crucially for surgical planning, neural elements in a single acquisition and without radiation exposure. Nevertheless, while sCT presents an exciting opportunity for the future, further research is needed to generate robust evidence for its accuracy and safety in both pre-operative planning and surgical navigation, ensuring its reliable clinical implementation.

This study has several limitations. The relatively small sample size and specific focus on distinct curve type and specific sex of patients undergoing pre-operative MRI scans may limit generalizability to the broad AIS population. Nevertheless, it is important to notice that the majority of AIS curves present within the main thoracic or thoracolumbar regions, and the deformity predominantly affects females [1, 2]. Therefore, our sample size covers most of the AIS population. Further research with a larger and broader population is important to reconfirm generalizability of sCT for scoliosis assessment. Additionally, while other studies suggest promising agreement between sCT and CT measurements in capturing complex structures, direct comparisons in this study were not possible due to absence of standard CTs [20, 22, 37]. The comparison of the standing angles with the calculated supine Cobb angles based on previously validated linear equations, however, demonstrated comparable results to previous studies. However, the comparison of supine sCT measurements with standing radiographic Cobb angles may introduce variability, as positional differences between supine and standing imaging can influence curve magnitude, contributing to the variability observed in the Bland–Altman analysis. [25]. Lastly, this proof-of-concept study does not include mild and more flexible curves and proof of its value in such cases cannot be confirmed. Nevertheless, sCT demonstrates high potential in preoperative cases which is the focus of this novel technology.

One strength of this study lies in its focus on utilizing sCT to evaluate scoliosis-specific measurements. sCT offers potential advantages over traditional methods, including accuracy in capturing complex 3D deformities and in parallel eliminating exposure to ionizing radiation [18, 20]. Additionally, the high inter-observer reliability demonstrated in this study supports the potential for consistent sCT image interpretation, strengthening its value as a potential clinical tool. Moreover, the measurements found in the sCT compared to the upright Cobb presented strong correlation with previous literature, confirming the accuracy [25]. While further research is warranted, these strengths suggest that sCT could play a valuable role in managing AIS by assisting in treatment decisions and pre-operative planning, potentially allowing for precise (surgical) intervention without radiation exposure. This could ultimately lead to improved patient outcomes and quality of life.

## Conclusions

This pilot study demonstrates the feasibility of sCT derived from MRI to provide radiation-free and reliable measurements of 3D deformity parameters in pre-operative AIS patients. While further research is needed for specific clinical applications, these findings confirm the potential for high-quality imaging of the bone morphology in AIS and its possible application in surgical treatment planning and potentially navigated surgery.

## Data Availability

As the data are privacy-sensitive, data will be available under restriction by sending an email to the corresponding author.
